# Seroprevalence of Hepatitis E Virus infection among volunteer blood donors in central province of Iran in 2012

**Published:** 2013-06

**Authors:** Hassan Ehteram, Amitis Ramezani, Ali Eslamifar, Masoomeh Sofian, Mohammad Banifazl, Shahin Ghassemi, Arezoo Aghakhani, Parisa Mashayekhi

**Affiliations:** 1 Department of Pathology, Faculty of Medicine, Kashan University of Medical Sciences, Kashan, Iran; 2 Departments of Clinical Research, Pasteur Institute of Iran, Tehran, Iran; 3 Tuberculosis and Pediatric Infectious Research Center, Arak University of Medical Sciences, Arak, Iran; 4 Iranian Society for Supporting Patients with Infectious Diseases, Tehran, Iran; 5 Firoozgar Hospital, Tehran University of Medical Sciences, Tehran, Iran; 6 Departments of Vacination, Pasteur Institute of Iran, Tehran, Iran

**Keywords:** Hepatitis E virus (HEV), Seroprevalence, Blood donor

## Abstract

**Background and Objectives:**

Hepatitis E virus (HEV) is a major public health concern in developing countries. HEV transmission occurs primarily by the fecal-oral route. It has also been reported that blood donors are potentially able to cause transfusion-associated hepatitis E in endemic areas. This study aimed to determine the seroprevalence of HEV infection among volunteer blood donors in Central province of Iran in 2012.

**Material and Methods:**

A total of 530 consecutive blood donor samples collected from Blood Transfusion Organization, Central Province of Iran. All samples were tested for the presence of IgG Hepatitis E antibody (anti-HEV) using enzyme-linked immunosorbent assay (ELISA).

**Results:**

From 530 blood donors, 91.9% were male and 8.1% were female. Overall, anti-HEV was found in 76 of 530 samples (14.3%). There was no significant difference in HEV seropositivity between the subjects regarding gender and area of residence (urban vs. rural). Anti-HEV was distributed among all age groups. Although people aged 31-50 years had the highest prevalence, but there was no statistical difference between the age groups.

**Conclusion:**

This study shows a relatively high prevalence of anti-HEV in the blood donors of Central province of Iran. More investigations are needed to assess the potential benefit of adding HEV screening of blood products to the current blood donor selection criteria.

## INTRODUCTION

Hepatitis E virus (HEV) appears to be the second most frequent cause of enteric hepatitis after hepatitis A virus infection (1). Some studies showed that HEV hepatitis is a major public health concern in developing countries (2–4).

HEV is an unclassified nonenveloped virus belongs to genus Hepevirus of the family Hepeviridae (3, 5). Its genome is a single-stranded, positive-sense RNA of approximately 7.2 kb (5). HEV isolates are classified into five major genotypes which belong to one serotype (6). Genotypes 1 and 2 exclusively infect humans and are endemic in many parts of Asia, Africa and South America and often associated with outbreaks and epidemics in developing countries (7–9). Genotypes 3 and 4 infect humans, pigs and other animal species and have been responsible for sporadic cases of disease. Genotype 5 infects avian species (7–9).

Transmission of HEV occurs primarily by the fecal-oral route through fecal contamination of drinking water in developing countries. HEV may also be transmitted parenterally as well as vertically particularly in endemic areas (10), but person to person transmission is uncommon (1). Recent studies have indicated that zoonosis is involved in the transmission of HEV, especially in industrialized countries (11, 12). It has also been reported that blood donors are potentially able to cause transfusion-associated hepatitis E in high endemic areas (13, 14).

The prevalence of HEV antibodies (anti-HEV) has been described in different populations. Iran is an endemic country for hepatitis E disease and its seroprevalence increased significantly with age, from 3.3% in subjects less than 30 years of age to 37.5% in individuals of 50 years (15, 16). A population-based study indicated that the prevalence rate of anti-HEV IgG among healthy population was 9.6% (17).

Providing a safer blood and blood products is a major concern of blood banks in the world. HEV infection is emerging as a potential new threat to blood safety after several cases of transfusion-transmission were reported from different countries (18–20). HEV is endemic in Iran; however limited data are available for HEV seroprevalence in blood donors of different parts of the country (2, 15, 21, 22). This study aimed to determine the seroprevalence of HEV infection among volunteer blood donors in Central province of Iran in 2012.

## MATERIALS AND METHODS

In this cross-sectional study, blood samples of 530 volunteer blood donors residing in urban and rural areas of Central province of Iran were collected consecutively from Iranian Blood Transfusion Organization in September 2012. Informed consent was obtained from all cases. The study was approved by Iranian Society for Support Patients with Infectious Diseases Ethics Committee.

Plasma samples were tested for IgG Hepatitis E antibody (anti-HEV) using enzyme-linked immunosorbent assay (ELISA) test.

Anti-HEV was detected by Dia.Pro Diagnostic BioProbes, Milan, Italy ELISA kit. This assay uses HEV-specific synthetic antigens derived from open reading frame (ORF) 2 and ORF3 of all 4 HEV subtypes. The procedure was followed as indicated by the manufacturer. Positive and negative controls were included in all the ELISA microplates assays. The anti-HEV detection sensitivity and specificity were 100%.

### Statistical Analysis

The Chi-square were used with the SPSS 16 Package program for statistical analysis (Chicago, IL, USA). A p-value of <0.05 was considered significant. Data was presented as mean ± SD or, when indicated, as an absolute number and percentage.

## RESULTS

A total of 530 volunteer blood donors were enrolled in the study. Of the study subjects, 91.9% were male and 8.1% were female. Their age ranged from 18 to 71 years with mean age 36.3 ± 11.7 years. 195 donors (36.8%) were aged 18-30 years, 266 cases (50.2%) were aged 31-50 years and 69 (13%) were older than 50 years. 10.4% of cases were habitant in rural areas and the remaining were resident in urban areas.

76 blood donors showed positive anti-HEV IgG antibodies, corresponding to an overall seroprevalence rate of 14.3%. There was no significant difference in HEV seropositivity between the subjects regarding gender (89.5% in males and 10.5% in females, p = 0.37). No significant differences were detected between the prevalence of anti-HEV antibodies in rural (6.6%) and urban (93.4%) areas (p = 0.24).

Anti-HEV was distributed among all age groups. Although people aged 31-50 years had the highest prevalence, but there was no statistical difference between the age groups (p = 0.3). General characteristics of the 530 examined blood donors are summarized in Table 1.

**Table 1:** Characteristics of the study population.

Variables	All donors n: 530	Anti-HEV positive n: 76	Anti-HEV negative n: 454
**Sex**			
Male	487 (91.9%)	68 (89.5%)	419 (92.3%)
Female	43 (8.1%)	8 (10.5%)	35 (7.7%)
**Area of residence**			
Urban	475 (89.6%)	71 (93.4%)	404 (89%)
Rural	55 (10.4%)	5 (6.6%)	50 (11%)
**Age group**			
18-30	195 (36.8%)	28 (36.9%)	167 (36.8%)
31-50	266 (50.2%)	34 (44.7%)	232 (51.1%)
>50	69 (13%)	14 (18.4%)	55 (12.1%)

Data indicated as absolute number (%); HEV: Hepatitis E Virus

## DISCUSSION

HEV is an important cause of epidemic hepatitis in developing countries. HEV hepatitis also occurs sporadically in some developed countries. The main transmission route of HEV is fecal-oral specially in endemic areas, but other transmission routes like parenteral exposure, vertical transmission, blood transfusion and dialysis were also reported, which are more common in non endemic areas (7, 23–25).

Iran is an endemic country for hepatitis E disease (26). Previous studies have demonstrated a variety of seroprevalence rates in different parts of Iran. In a population-based study conducted by Taremi et al. (17) the prevalence of anti-HEV IgG among healthy population in Iran was reported as 9.6%. Another general survey in Mazendaran (North of Iran) showed that 1.1% of children younger than 10 years old and 7.2% of population between 20-25 years old had anti-HEV IgG (27). Another study from this area reported that the prevalence of HEV between adults was 11.8% (28). Ataei et al. (1) showed 3.8% anti-HEV seroprevalence rate in Isfahan province. They reported that HEV seroprevalence increased with age from 0.9% in children aged 6-9 years to 8.1% in people over 50 years old. A recent study in general population of Tehran demonstrated that IgG anti-HEV seroprevalence rate was 9.3% (29).

The results of few studies on blood donors in Iran showed different seroprevalence rates. In two independent performed studies, the prevalence of HEV was 12.9% in Hamedan province (30) and 7.8% in Tabriz city, East Azerbaijan Province (31). Another investigation in Khuzestan Province (Southwest Iran) showed that 11.5% of urban healthy blood donors had IgG anti-HEV (21). In two different studies on blood donors of Tehran, anti-HEV was detected in 7.8% and 4.5% of cases, respectively (2, 22).

The HEV seroprevalence in blood donors in other countries were reported as 0.95% to 20.6% in Europe (32, 33), 2.3% in Brazil (34), 3.7% in Japan (5), 32.6% in China (35) and 4% to 45.2% in the Middle East (36, 37).

In this study we investigated the seroprevalence of HEV infection among volunteer blood donors in Central province of Iran. Anti-HEV was detected in 14.3% of the cases. So the prevalence of anti-HEV in current study is higher than reported in previous studies from other parts of Iran (2, 21, 22, 30, 31) and in the range of other studies in the Middle East (36, 37). It can be due to differences in the demographics of studied population, the size of the samples, the public health services situation and used anti-HEV detection assays. Because HEV-RNA was not examined in our study, we cannot draw conclusions regarding active HEV prevalence in our blood donors. As both HEV epidemiology and cost effectiveness of a test are important in deciding whether adding a screening test to current blood product screening tests is appropriate or not, this topic merits further study.

In conclusion, this study shows a relatively high prevalence of anti-HEV in the blood donors of Central Province of Iran. Further studies on HEV prevalence rates in blood donors in different parts of the country are required to evaluate the safety of blood products and potential benefit of HEV screening in blood banks.
